# Health Care Resource Utilization for Esophageal Cancer Using Proton versus Photon Radiation Therapy

**DOI:** 10.14338/IJPT-22-00001.1

**Published:** 2022-06-23

**Authors:** Steven H. Lin, Kaiping Liao, Xiudong Lei, Vivek Verma, Sherif Shaaban, Percy Lee, Aileen B. Chen, Albert C. Koong, Wayne L. Hoftstetter, Steven J. Frank, Zhongxing Liao, Ya-Chen Tina Shih, Sharon H. Giordano, Grace L. Smith

**Affiliations:** 1 Department of Radiation Oncology, The University of Texas MD Anderson Cancer Center, Houston, TX, USA; 2 Department of Health Services Research, The University of Texas MD Anderson Cancer Center, Houston, TX, USA; 3 Department of Thoracic and Cardiovascular Surgery, The University of Texas MD Anderson Cancer Center, Houston, TX, USA

**Keywords:** proton therapy, intensity-modulated radiation therapy, esophageal cancer, resource utilization, postoperative complications

## Abstract

**Purpose:**

In patients treated with chemoradiation for esophageal cancer (EC), randomized trial data demonstrate that proton beam therapy (PBT) reduces toxicities and postoperative complications (POCs) compared with intensity-modulated radiation therapy (IMRT). However, whether radiation therapy modality affects postoperative health care resource utilization remains unknown.

**Materials and Methods:**

We examined 287 patients with EC who received chemoradiation (prescribed 50.4 Gy/GyE) followed by esophagectomy, including a real-world observational cohort of 237 consecutive patients treated from 2007 to 2013 with PBT (n = 81) versus IMRT (n = 156); and an independent, contemporary comparison cohort of 50 patients from a randomized trial treated from 2012 to 2019 with PBT (n = 21) versus IMRT (n = 29). Postoperative complications were abstracted from medical records. Health care charges were obtained from institutional claims and adjusted for inflation (2021 dollars). Charge differences (Δ = $PBT − $IMRT) were compared by treatment using adjusted generalized linear models with the gamma distribution.

**Results:**

Baseline PBT versus IMRT characteristics were not significantly different. In the observational cohort, during the neoadjuvant chemoradiation phase, health care charges were higher for PBT versus IMRT (Δ = +$71,959; 95% confidence interval [CI], $62,274–$82,138; *P* < .001). There was no difference in surgical charges (Δ = −$2234; 95% CI, −$6003 to $1695; *P* = .26). However, during postoperative hospitalization following esophagectomy, health care charges were lower for PBT versus IMRT (Δ = −$25,115; 95% CI, −$37,625 to −$9776; *P* = .003). In the comparison cohort, findings were analogous: Charges were higher for PBT versus IMRT during chemoradiation (Δ = +$61,818; 95% CI, $49,435–$75,069; *P* < .001), not different for surgery (Δ = −$4784; 95% CI, −$6439 to $3487; *P* = .25), and lower for PBT postoperatively (Δ = −$27,048; 95% CI, −$41,974 to −$5300; *P* = .02). Lower postoperative charges for PBT were especially seen among patients with any POCs in the contemporary comparison (Δ = −$176,448; 95% CI, −$209,782 to −$78,813; *P* = .02).

**Conclusion:**

Higher up-front chemoradiation resource utilization for PBT in patients with EC was partially offset postoperatively, moderated by reduction in POC risks. Results extend existing clinical evidence of toxicity reduction with PBT.

## Introduction

Neoadjuvant chemoradiation (nCRT) plus esophagectomy is standard curative treatment for esophageal cancer (EC) [1–3]. This trimodality approach, however, is associated with postoperative complications (POCs), including major cardiac and pulmonary adverse events [4]. Prior clinical studies of patients with EC have found that radiation modality approaches could affect postoperative pulmonary complications [5], and that proton beam therapy (PBT) is associated with a lower frequency of major organ POCs compared with photon-based radiation therapy [6], an outcome attributed to the favorable dosimetric profile of protons that decreases radiation dose to normal organs including the heart and lungs [7, 8]. A recent randomized trial that included patients with EC treated with trimodality or bimodality (definitive chemoradiation) treatment demonstrated a lower toxicity burden and markedly reduced POCs with PBT compared with intensity-modulated radiation therapy (IMRT) [9].

Despite the favorable findings for toxicity risks after PBT for patients with EC, one major barrier to wider acceptance of PBT as a standard option for EC is that delivery costs of PBT exceed that of IMRT— along with additional capital investment, quality assurance, and operations costs [10]. As a result, the comparative effectiveness of PBT for EC within a value framework has remained undefined, despite increasing recognition of the need to advance value-based oncologic decision-making in cancer care delivery [11, 12]. Specifically, it is unknown whether up-front costs and resource utilization for delivering PBT could be offset by lower health care resource utilization in the postoperative phase, driven by reductions in POCs. Even though models in prior studies showed that the potential toxicity-sparing effects of PBT could potentially result in resource savings for some tumor types [13, 14], high-quality data on resource utilization is currently lacking for EC [15].

To address this knowledge gap, we sought to examine the resource utilization associated with curative-intent PBT-based versus IMRT-based trimodality therapy for EC. To do so, first, we examined health care charges through the phases of curative treatment in an observational cohort of “real-world” patients receiving trimodality treatment for EC. Second, we sought to compare findings in an independent, contemporary cohort of patients with EC randomized to treatment with PBT- versus IMRT-based trimodality therapy in the setting of a clinical trial (NCT01512589) [9]. Third, we sought to characterize the potential moderating factors of postoperative resource utilization in patients treated with PBT-based versus IMRT-based therapy.

## Materials and Methods

### Study Sample

**Figure 1.**
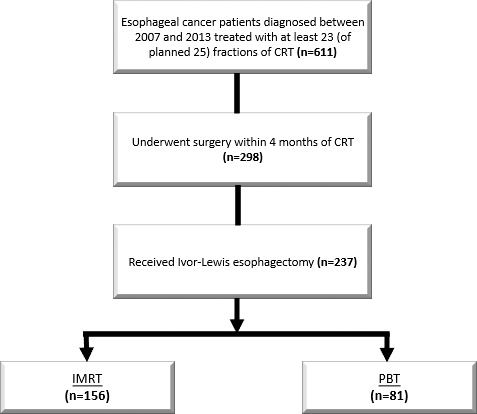
Observational cohort: inclusion criteria for analysis.

This analysis was approved by the University of Texas MD Anderson Cancer Center institutional review board. This study examined 2 independent patient cohorts. An observational cohort was drawn from a retrospective population of 611 consecutive patients with EC treated at our institution with nCRT (completing at least 23 fractions of the prescribed radiation prescription of 50.4 Gy or GyE in 28 fractions) from 2007 to 2013, with the selected time period representing care delivery prior to uniform institutional implementation of an enhanced recovery after surgery (ERAS) protocol. Among esophageal surgical patients, this protocol affected inpatient postoperative management, resource utilization, and clinical outcomes. This cohort was also treated before comprehensive implementation of the aforementioned institutional randomized trial [16, 17]. Of the 611 patients, 298 received surgery within 4 months of nCRT, and among these, 237 received the most common surgical approach of Ivor-Lewis esophagectomy; these 237 patients were included in the analytic sample (**Figure 1**).

The independent, contemporary comparison cohort was drawn from 107 patients who were enrolled in the published randomized trial of PBT versus IMRT for EC from our institution from 2012 to 2019, among whom 51 received trimodality therapy [9]. Excluded was 1 patient who received salvage surgery more than 1 year from IMRT completion, for a total sample of 50 patients included in the comparison cohort analysis. Complete details of the randomized trial design and protocol are in its original publication [9]. Patients in this trial were prescribed the same radiation treatment as in the observational cohort (50.4 Gy or GyE in 28 fractions) in the present analysis. Per trial protocol, the surgical technique was per surgeon discretion. Analysis of health care charges was a prespecified secondary objective of this protocol.

### Outcomes and Covariates

Health care charges were obtained from institutional claims data on each patient, tabulating all charges associated with 3 phases of cancer therapy: (1) the nCRT phase (an aggregate of all technical and professional charges pertaining to chemotherapy and radiotherapy, from the first to the last date of nCRT), (2) the day of surgery (an aggregate of all charges that occurred from when the patient was admitted for surgery until the patient left the operating room), and (3) postoperatively (an aggregate of charges between leaving the operating room until hospital discharge). Adjustment for inflation was performed [11] by normalizing charges to 2021 US dollars.

For this analysis, in each cohort, radiation treatment was categorized as PBT versus IMRT as identified from the medical record. Additional demographic and clinical covariates, including patient age, sex, race, cancer stage, performance status, and comorbidities were abstracted from the medical record. Postoperative complications abstracted from the medical record included pulmonary (pneumonia, pleural effusion, chylothorax, pulmonary embolism, acute respiratory distress syndrome, or respiratory insufficiency); cardiac (including new onset atrial fibrillation, atrial or ventricular arrhythmias, myocardial infarction, or congestive heart failure); and gastrointestinal (GI) events (including anastomotic leak, ileus, fistula, bowel obstruction, or necrosis).

### Statistical Analysis

Chi-square tests compared patient characteristics between treatment groups in the observational cohort. In both cohorts, charges for PBT versus IMRT groups were compared using generalized linear modeling with the gamma distribution (accounting for skewness) [18]. Comparisons were expressed as absolute differences (Δ = $PBT − $IMRT). To characterize the comparison as a relative difference, the difference was also presented as a charge ratio (CR; $PBT/$IMRT), given a historical framework of assessing particle therapy costs or charges using ratios [10]. Multivariable generalized linear regression models were adjusted for covariates. We included a priori age, sex, and performance status as covariates, and in models for the comparison cohort, included tumor-location and surgery-type covariates owing to statistical significance (*P* < .05). Charge ratios were analyzed for individual strata (eg, age ≤44, 45–54, 55–64, 65–74, ≥75 years; 0, 1 ≥2 POCs). To identify the effect of age and POCs on postoperative charges, interaction terms with radiation treatment type was also tested. The cost-to-charge calculation is based on the average cost ratios for urban hospitals in Texas for Medicare reimbursements, which is set at 0.28 as the multiplier [19, 20]. All *P* values were 2-sided, with a threshold of .05 to determine significance. Analyses were conducted using SAS version 9.4 (SAS Inc, Cary, North Carolina).

## Results

### Patient Characteristics

Of 237 patients in the observational cohort, 156 patients received IMRT (66%) and 81 received PBT (34%). There were no statistically significant differences in clinical or demographic characteristics between patients in the 2 treatment groups, including age, race, sex, and performance status (**Table 1**). In this group, the overall median age was 61 years (interquartile range [IQR], 54–68), 89% were men, and 40% had Karnofsky performance status score of <90.

**Table 1.** Observational cohort characteristics for patients with esophageal cancer.

	**Total N**	**IMRT, N (%)**	**PBT, N (%)**	***P*** **value**
Age (y)				.62
≤44	24	17 (70.83)	7 (29.17)	
45–54	48	31 (64.58)	17 (35.42)	
55–64	80	57 (71.25)	23 (28.75)	
65–74	70	42 (60.00)	28 (40.00)	
75+	15	9 (60.00)	6 (40.00)	
Sex				.93
Female	24	16 (66.67)	8 (33.33)	
Male	213	140 (65.73)	73 (34.27)	
Race				.55
White	219	143 (65.30)	76 (34.70)	
Non-white	18	13 (72.22)	5 (27.78)	
Cancer stage				.88
I–II	92	60 (65.22)	32 (34.78)	
III–IV	145	96 (66.21)	49 (33.79)	
Karnofsky performance status				.49
<90	98	67 (68.37)	31 (31.63)	
≥90	139	89 (64.03)	50 (35.97)	
Comorbidities				
Hypertension				.10
No	111	79 (71.17)	32 (28.83)	
Yes	126	77 (61.11)	49 (38.89)	
Asthma				.41
No	231	153 (66.23)	78 (33.77)	
Yes	6	3 (50.00)	3 (50.00)	
Reflux				.67
No	142	95 (66.90)	47 (33.10)	
Yes	95	61 (64.21)	34 (35.79)	
Diabetes				.16
No	201	136 (67.66)	65 (32.34)	
Yes	36	20 (55.56)	16 (44.44)	
Coronary artery				.26
No	212	137 (64.62)	75 (35.38)	
Yes	25	19 (76.00)	6 (24.00)	
CABG				.67
No	231	151 (65.37)	80 (34.63)	
Yes	6	5 (83.33)	1 (16.67)	
AF				1.00
No	229	151 (65.94)	78 (34.06)	
Yes	8	5 (62.50)	3 (37.50)	
COPD				.38
No	224	149 (66.52)	75 (33.48)	
Yes	13	7 (53.85)	6 (46.15)	

**Abbreviations:** IMRT, intensity-modulated radiation therapy; PBT, proton beam therapy; CABG, coronary artery bypass graft; AF, atrial fibrillation; COPD, chronic obstructive pulmonary disease.

Of 50 patients in the contemporary comparison cohort, 29 (58%) underwent IMRT, and 21 (42%) received PBT. Complete characteristics of these patients are published in the results from the primary trial and similarly demonstrated no difference in age, race, sex, comorbidity, chemotherapy, and surgery type by treatment group [6]. In this group, the median age was 65.5 years (IQR, 59–70), 86% were men, and 32% had Karnofsky performance status score of <90. A total of 64% were treated with Ivor-Lewis esophagectomy and 24%, with minimally invasive esophagectomy.

### Observational Cohort: Health Care Resource Utilization across Phases of Care

**Figure 2.**
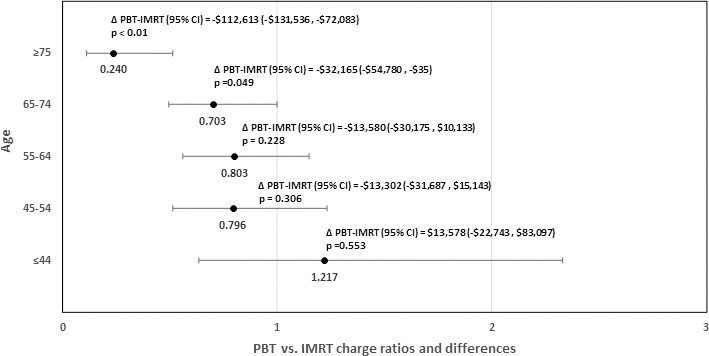
Charges in patients receiving proton beam therapy (PBT) versus intensity-modulated radiation therapy (IMRT) in the postoperative phase, using charges per day during admission, stratified by patient age group among esophageal cancer patients in the observational cohort (N = 237).

During the nCRT phase of care, adjusted health care charges were higher for patients treated with PBT relative to IMRT (Δ = +$71,959; 95% CI, $62,274–$82,138; CR = 1.56; 95% CI, 1.48–1.64; *P* < .001). For esophagectomy, there was no difference in surgical charges for patients treated with PBT versus IMRT (Δ = −$2234; 95% CI, −$6003 to $1695; CR = 0.98; 95% CI, 0.94–1.02; *P* = .26). However, there were potential savings in patients treated with PBT during the postoperative inpatient admission phase of care, with total postoperative charges significantly lower in PBT versus IMRT (Δ = −$25,115; 95% CI, −$37,625 to −$9776; CR = 0.73; 95% CI, 0.60–0.89; *P* = .003). See also **Table 2** for details, including unadjusted estimates. In the observational cohort, age was a modifying factor in the postoperative phase (**Figure 2**).

**Table 2.** Adjusted and unadjusted health care charges (in 2021 dollars) by phase of care in observational and contemporary comparison cohorts for patients with esophageal cancer treated with PBT versus IMRT.

	**Unadjusted**			**Adjusted**		
	**Δ PBT − IMRT, charge $ (95% CI)**	**CR: PBT/IMRT (95% CI)**	***P*** **value**	**Δ PBT − IMRT, charge $ (95% CI)**	**CR: PBT/IMRT (95% CI)**	***P*** **value**
Observation cohort^a^						
nCRT	69,671 (60,265 to 79,540)	1.56 (1.48 to 1.64)	<.001	71,959 (62,274 to 82,138)	1.56 (1.48 to 1.64)	<.001
Surgery Day	−1,995 (−5,761 to 1,933)	0.98 (0.94 to 1.02)	.315	−2,234 (−6,003 to 1,695)	0.98 (0.94 to 1.02)	.26
Postoperative	−20,525 (−32,180 to −6,184 )	0.75 (0.61 to 0.93)	.007	−25,115 (−37,625 to −9,776)	0.73 (0.60 to 0.89)	.003
Comparison cohort^b^						
nCRT	62,632 (49,606 to 76,622 )	1.5 (1.40 to 1.61)	<.001	61,818 (49,435 to 75,069)	1.49 (1.39 to 1.60)	<.001
Surgery Day	−3,152 (−13,129 to 8,114)	0.97 (0.85 to 1.09)	.57	−4,784 (−6,439 to 3,487)	0.95 (0.86 to 1.04)	.25
Postoperative	−52,518 (−69,569 to −25,6495)	0.47 (0.30 to 0.74)	.002	−27,048 (−41,974 to −5,300)	0.64 (0.44 to 0.93)	.02

**Abbreviations:** PBT, proton beam therapy; IMRT, intensity-modulated radiation therapy; CR, charge ratio; CI, confidence interval; nCRT, neoadjuvant.

a Model adjusted for age, sex, and performance status.

b Model adjusted for age, sex, performance status, tumor location, and surgery type.

### Contemporary Comparison Cohort: Health Care Resource Utilization across Phases of Care

In the comparison cohort, there were analogous findings for health care charges across phases of care. During the nCRT phase, adjusted health care charges were higher for patients treated with PBT relative to IMRT (Δ = +$61,818; 95% CI, $49,435–$75,069; CR = 1.49; 95% CI, 1.39–1.60; *P* < .001). For esophagectomy, there was no significant difference in surgical charges for patients treated with PBT versus IMRT (Δ = −$4784; 95% CI, −$6439 to $3487; CR = 0.95; 95% CI, 0.86–1.04, *P* = .25). Still, postoperative charges were significantly lower in patients treated with PBT versus IMRT (Δ = −$27,048; 95% CI, −$41,974 to −$5300, CR = 0.64; 95% CI, 0.44–0.93; *P* = .02). See also **Table 2** for details, including unadjusted estimates. In this cohort, older age (dichotomized at 65 years) did not modify postoperative charges (P_interaction_ = 0.97).

### Postoperative Complications

Postoperatively in the observational cohort, pulmonary complications were the most common (18%), followed by GI (16%), and cardiac (9%). Developing any complications was significantly associated with longer hospital stay (*P* < .0001) (**Supplemental Table S1**). Patients who developed a single postoperative complication of any type (n = 59, 25%) and those with 2 or more complications (n = 21, 9%) had significantly longer hospital stay than patients without complications (*P* < .0001) (**Supplemental Table S2**). While we found only a trend of higher rates of pulmonary and cardiac complications in IMRT, the mean length of stay in the IMRT group (10.4 days) was significantly longer than the PBT group (8.4 days) (*P* < .0001) (**Supplemental Table S2**). The comparison cohorts had similarly higher but nonsignificantly increased risk in POCs, as well as a strong trend toward higher hospitalizations in the IMRT group (**Supplemental Table S3**).

A higher burden of POCs, particularly multiple (≥2) POCs, occurred more frequently in patients treated with IMRT than PBT. This difference was statistically significant among patients in the comparison cohort (**Table 3**). Results also supported a moderating effect of POCs on health care resource utilization in the postoperative setting, with higher postoperative savings with PBT especially within the stratum of patients who experienced any POC in the observational cohort (Δ = −$37,329; 95% CI, −$63,971 to −$1250; *P* = .04) and the contemporary comparison cohort (Δ = −$176,448; 95% CI, −$209,782 to −$78,813; *P* = .02).

**Table 3.** Postoperative complications and health care charges in patients with esophageal cancer treated with proton beam therapy versus intensity-modulated radiation therapy within the observational and contemporary comparison cohorts.

	**Observation (N = 237)**			**Comparison (N = 50)**		
	**IMRT, N (Column %)**	**PBT, N (Column %)**	***P*** **value**	**IMRT, N (Column %)**	**PBT, N (Column %)**	***P*** **value**
Number of POC						
0	102 (65.4)	55 (67.9)	.30	20 (69.0)	18 (85.7)	<.001
1	37 (23.7)	22 (27.2)		2 (6.9)	3 (14.3)	
2+	17 (10.9)	4 (4.9)		7 (24.1)	0 (0)	
Stratified by POC						
No POC						
Δ PBT − IMRT (95% CI)	−$9,374 (−$17,645 to $854)		.07	$4,370 (−$6,746 to $13,268)		.48
CR (95% CI)	0.82 (0.67 to 1.02)			1.11 (0.84 to 1.46)		
Any POC						
Δ PBT − IMRT (95% CI)	−$37,329 (−$63,971 to −$1,250)		.04	−$176,448 (−$209,782 to −$78,813)		.02
CR (95% CI)	0.73 (0.54 to 0.99)			0.22 (0.08 to 0.65)		

**Abbreviations:** PBT, proton beam therapy; IMRT, intensity-modulated radiation therapy; POC, postoperative compliations; CR, charge ratio; CI, confidence interval.

## Discussion

Our study of patients with EC demonstrated that, though initial health care resources to deliver PBT were greater than for IMRT, a portion of the higher charges associated with PBT incurred during nCRT phase of care were offset by lower charges within the immediate postoperative window, driven strongly by burden of complications. As another perspective for framing in context, given that health care charges are greater than actual costs incurred, when this estimate is multiplied by a factor of 0.28 (the average Medicare cost-to-charge ratio for urban hospitals in the state [19, 20]), the estimated excess up-front direct costs incurred would translate to $17,309 and the estimated postoperative incurred costs offset would translate to −$7573.

Our results were robust within both a “real-world” treatment setting and a contemporary setting where treatment was delivered in the context of a randomized clinical trial. The analysis spanned the period from 2007 to 2019, with a similar magnitude of adjusted relative and absolute postoperative savings with PBT, regardless of the study setting and the time period. Results further supported that POCs represent an important potential moderator of the association between neoadjuvant chemoradiation treatment modality and downstream postoperative resource utilization. Though POCs were slightly more frequent in the earlier era of the observational cohort, before wide uptake of enhanced perioperative management techniques, still, the moderating effect of burden of POCs was seen in the contemporary comparison cohort. Our findings are significant in the context of acute events representing one of the costliest components of cancer care delivery [21].

Our study's results are also significant in the context of the wider mounting evidence that PBT reduces major organ toxicity burden in patients with EC treated with chemoradiation, including the full randomized clinical trial (composed of surgically resected and unresected patients) from which our contemporary comparison cohort was derived [5, 6, 9]. While this assembling body of evidence supports a clinical benefit of PBT as a treatment option for EC, nevertheless, an oncology value framework needs to consider not only clinical outcomes and toxicity profile but also how treatment options differentially affect resource utilization across care [22–24]. Results from our analysis therefore contribute to understanding resource utilization with PBT for EC across multiple phases of care and add to the current understanding of quality of life [25] and toxicity [9] profile after PBT and to the context of known greater up-front costs to deliver PBT [10, 13, 26].

Up-front charges for delivery of PBT versus IMRT (approximately 50% greater) in this analysis were consistent with prior studies of PBT, reflecting relative uniformity of resource requirements for capital investment, operations, and PBT delivery [10]. However, our study demonstrates a novel finding of partially offset charges, for patients receiving PBT, during the period immediately following inpatient admission—occurring within the timeframe for high risk of acute major organ complications after surgery. To date, one of the most widely accepted benefits of PBT in providing value for cancer care is among pediatric patients who have high risk for long-term toxicities with radiation treatment [27, 28]. Until recently, the prevailing paradigm for the rationale for PBT, derived from this setting, argued for its particular advantage in young patients with long expected survival who benefit from long-term toxicity reduction. Under that paradigm, the optimal value of PBT was expected to emerge on the scale of years to decades after the radiation treatment was initially delivered.

In contrast, results from the present analysis support a separate, emerging rationale for PBT, with the value benefit of PBT attributed to health care resources partially recaptured in the short term—both by the individual patient as well as the health system—from acute toxicity reduction. In this paradigm of characterizing the value of PBT, PBT could offer particular advantage to patients with EC who are at highest risk of acute POCs—including such patients as the elderly, the frail, and those with underlying comorbidities [29, 30]. This paradigm is consistent with results of the noteworthy recent study by Baumann et al, who reported that among a large retrospective cohort of 1483 individuals with advanced cancer treated with chemoradiation, patients treated with PBT compared with patients treated with photons had fewer grade 2 to grade 3 toxicities within 90 days [31]. Results of our study provide a critical advance in the body of evidence on comparative effectiveness of PBT by addressing health care resource utilization.

Our study has several limitations. Patients for this analysis and the charges were derived from a single institution. Thus, the exact magnitude of cost findings may not reflect costs and charges at other institutions and within other economic systems globally. Charges accrued for health care resources used outside the institution also were excluded, though the examined phases of care represented periods of active oncology treatment. The multicenter NRG-GI006 trial (NCT03801876), currently accruing, may provide additional prospective multicenter data for validation once the trial completes [35]. Additionally, resource utilization for this study focused on immediate phases of care for radiation treatment delivery, surgery, and postoperative hospital admission but did not assess longer-term resource utilization (eg, 90-day postdischarge or lifetime costs). A potential modifying effect of ERAS was not directly analyzed in this study since it was uniformly implemented at an institutional level in the latter era represented during the randomized trial. Future studies may seek to quantify the effect of ERAS management on the relative effect on costs and resource utilization for different neoadjuvant therapy options.

## Conclusions

Despite higher initial technical and operational costs for PBT, the overall cost differential of PBT and IMRT may in fact be smaller than expected owing to reduced POCs and shorter hospital stay in the PBT group. So, while the costs for PBT are relatively higher up front, the clinical benefits may become apparent with time, namely, with respect to survival, late toxicities, and quality of life [32, 33]. Therefore, future cost-effectiveness studies are required to estimate the incremental lifetime costs of PBT that account for both treatment and posttreatment costs [34]. This can be performed as a secondary endpoint of the ongoing randomized study of PBT and IMRT and will provide further insight into economic evaluation of PBT for esophageal cancer treatment [34].
