# Supplementary material for: Health Care Resource Utilization for Esophageal Cancer Using Proton versus Photon Radiation Therapy
Source: Int J Part Ther. 2022 Jun 23;9(1):18–27. doi: 10.14338/IJPT-22-00001.1 (PMC9238132; doi:10.14338/IJPT-22-00001.1)
Supplement: Supplementary file 1 [file ijpt-09-01-09_s01.docx]

**Supplementary Tables**

**Table S1 – Overall Postoperative Complications and Length of Hospital Stay**

|  | **N** | **Mean** | **SD** | **Median** | **Min** | **Max** | **p-value** |  |
| --- | --- | --- | --- | --- | --- | --- | --- | --- |
| **Pulmonary** |  |  |  |  |  |  |  |  |
| **No** | 194 | 8.77 | 4.14 | 8 | 4 | 29 |  |  |
| **Yes** | 43 | 14.72 | 10.24 | 12 | 6 | 49 | <.0001 |  |
| **GI** |  |  |  |  |  |  |  |  |
| **No** | 199 | 9.46 | 6.11 | 8 | 4 | 49 |  |  |
| **Yes** | 38 | 11.87 | 6.12 | 9 | 5 | 28 | <.0001 |  |
| **Cardiac** |  |  |  |  |  |  |  |  |
| **No** | 216 | 9.50 | 5.84 | 8 | 4 | 49 |  |  |
| **Yes** | 21 | 13.48 | 8.16 | 11 | 6 | 35 | <.0001 |  |
| **Any Complication** |  |  |  |  |  |  |  |  |
| **No** | 157 | 8.23 | 3.42 | 8 | 4 | 29 |  |  |
| **Yes** | 80 | 13.03 | 8.66 | 10.5 | 5 | 49 | <.0001 |  |
| **No. of Complications** |  |  |  |  |  |  |  |  |
| **0** | 157 | 8.23 | 3.42 | 8 | 4 | 29 |  |  |
| **1** | 59 | 12.34 | 8.84 | 9 | 5 | 49 | <.0001 | 1 vs. 0 |
| **2+** | 21 | 14.95 | 8.04 | 12 | 7 | 35 | <.0001 | 2 vs. 0 |

**Table S2 – RT Adjusted Postoperative Complications and Length of Hospital Stay (LOS) in the Observational Cohort**

|  | **IMRT** | | | | **PBT** | | | | **p-value** |
| --- | --- | --- | --- | --- | --- | --- | --- | --- | --- |
|  | **No** | | **Yes** | | **No** | | **Yes** | |  |
|  | **N** | **%** | **N** | **%** | **N** | **%** | **N** | **%** |  |
| **Complication** |  |  |  |  |  |  |  |  |  |
| Pulmonary | 125 | 80.13 | 31 | 19.87 | 69 | 85.19 | 12 | 14.81 | 0.3380 |
| GI | 131 | 83.97 | 25 | 16.03 | 68 | 83.95 | 13 | 16.05 | 0.9962 |
| Cardiac | 141 | 90.38 | 15 | 9.62 | 75 | 92.59 | 6 | 7.41 | 0.5705 |
|  | **Avg. (SD)** | | | | **Avg. (SD)** | | | |  |
| **LOS** | 10.4 (6.3) | | | | 8.4 (5.7) | | | | <.0001 |

IMRT: Intensity-Modulated Radiation Therapy; PBT: Proton Beam Therapy

**Table S3 - RT Adjusted Postoperative Complications and Length of Hospital Stay (LOS) in the Comparison Cohort**

|  | **IMRT** | | | | **PBT** | | | | **p-value** |
| --- | --- | --- | --- | --- | --- | --- | --- | --- | --- |
|  | **No** | | **Yes** | | **No** | | **Yes** | |  |
|  | **N** | **%** | **N** | **%** | **N** | **%** | **N** | **%** |  |
| **Complication** |  |  |  |  |  |  |  |  |  |
| Pulmonary | 22 | 75.9 | 7 | 24.1 | 20 | 95.2 | 1 | 4.8 | 0.12 |
| GI | 27 | 93.1 | 2 | 6.9 | 21 | 100 | 0 | 0 | 0.50 |
| Cardiac | 28 | 96.6 | 1 | 3.4 | 21 | 100 | 0 | 0 | 1.0 |
|  | **Avg. (SD)** | | | | **Avg. (SD)** | | | |  |
| **LOS** | 13.0 (10.5) | | | | 9.1 (2.3) | | | | 0.06 |

IMRT: Intensity-Modulated Radiation Therapy; PBT: Proton Beam Therapy
